# Methods for studying the effects of phosphorylation patterns in proteins

**DOI:** 10.1042/BST20250137

**Published:** 2026-03-13

**Authors:** Shachar Guy Bressler, Dana Grunhaus, Mattan Hurevich, Assaf Friedler

**Affiliations:** The Institute of Chemistry, The Hebrew University of Jerusalem, Edmond J. Safra Campus, Givat Ram, Jerusalem 91904, Israel

**Keywords:** multiphosphorylation, peptides, phosphorylation, proteins

## Abstract

Protein phosphorylation is one of the most common and versatile regulatory mechanisms in cells. Most human proteins are phosphorylated at multiple sites, giving rise to large numbers of possible phosphorylation patterns. Each phosphorylation pattern can lead to a different functional or pathological outcome. Yet, linking defined phosphorylation patterns to specific biological functions remains a major experimental challenge. In this review we describe the main strategies to study phosphorylation patterns at the protein and domain levels and highlight how they complement each other. We first discuss cellular approaches, including phosphomimetics, kinase-based assays, and genetic code expansion, which allow working in a native environment but have their significant drawbacks. We then describe *in vitro* methods, such as enzymatic phosphorylation and semi-synthetic phosphoproteins generated by ligation, which afford mechanistic insights but result in low yields and are difficult to scale for producing libraries. We focus on synthetic phosphopeptide libraries as tools that offer precise control over the number and position of phosphosites and are uniquely suited for systematic mapping of phosphorylation patterns. This comes at a price of not working at the protein level, but rather at the domain level. Peptide libraries are often used for preliminary identification of key phosphorylations, later studied in detail at the protein level. We conclude that ideally more than one method should be used and that these methods should not be viewed as competing but rather as complementary. A combined use of several of these approaches provides a practical toolbox for dissecting how phosphorylation patterns regulate protein behavior.

## Phosphorylation patterns regulate protein function

Post-translational modifications (PTMs) are widespread regulators of protein structure, dynamics, and function. Phosphorylation of threonine, serine, or tyrosine is one of the most prevalent and extensively studied PTMs. Approximately 75% of all human proteins are phosphorylated, and most of these are modified at multiple sites [1]. Among the phosphorylated proteins, 27% undergo phosphorylation at one or two sites, 44% undergo phosphorylation at 3–12 sites, and 26% of the proteins can be phosphorylated at more than 12 identified sites [2–4], with an average of around seven phosphorylations per protein [5]. The complexity introduced by phosphorylation is enormous: a protein with just three potential phosphorylation sites will have eight distinct phosphorylation patterns, of different combinations of phosphorylated sites. Each such form can lead to a different protein functionality or biological outcome. With 16 possible phosphorylation sites, the Rb protein exceeds ∼2 ×10^13^ possible phosphorylation patterns [6]. When the number of sites increases to 40, like in the Tau protein [7], the number of potential phosphorylation patterns reaches an astronomical number of ∼8 × 10^47^. Experimentally, *in vitro*, Tau was found to adopt only 12–40 distinct experimentally observed phosphorylation patterns [8]. This means that there is a regulatory bias, and not every potential phosphorylation pattern is formed.

## The chemical properties of the phosphate group in phosphoproteins

**Figure 1:**
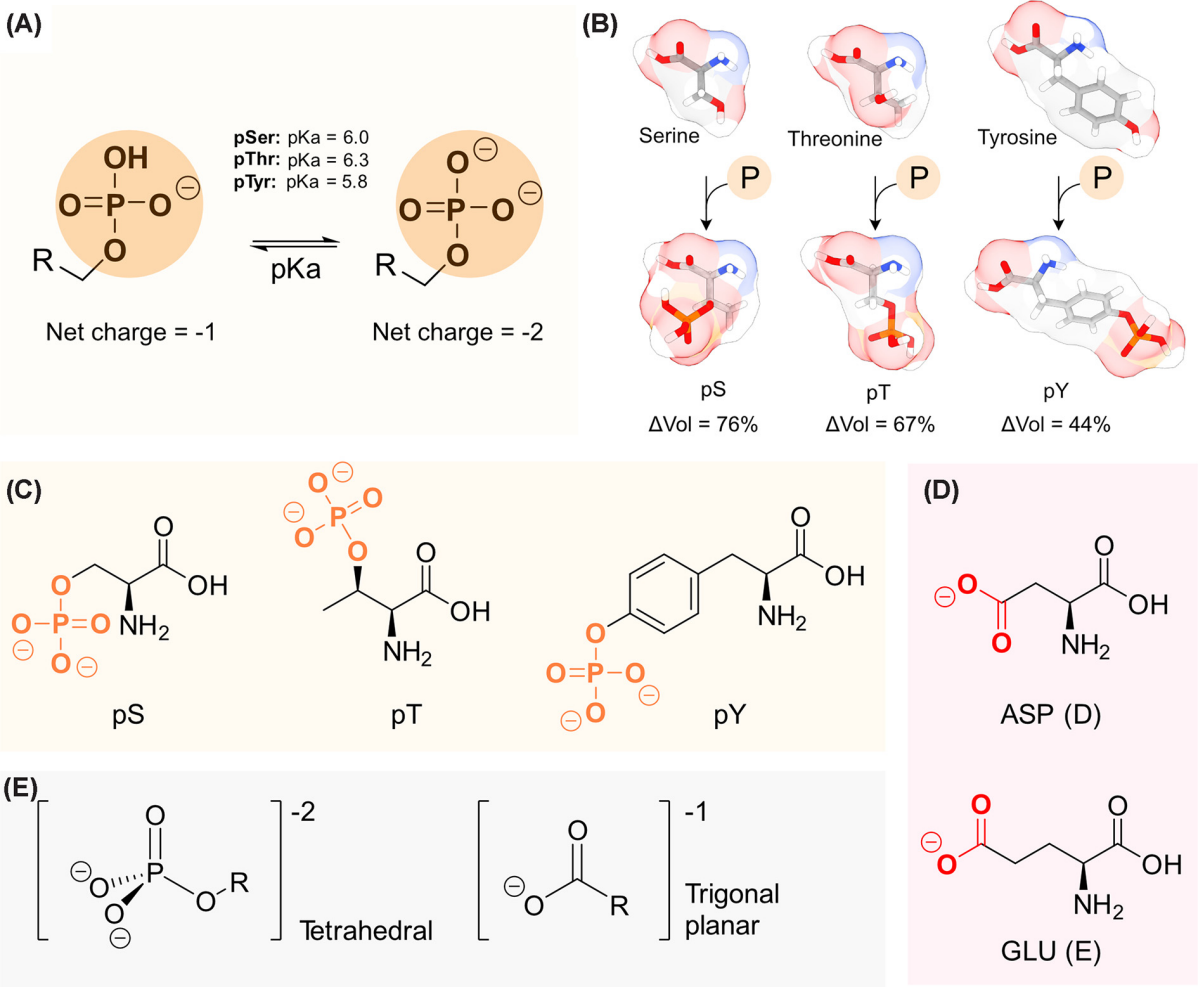
The chemical properties of phosphate group (**A**) The second pKa values of phosphorylated residues, which are close to physiological pH. (**B**) Volume differences between serine, threonine, and tyrosine (top row) and their phosphorylated derivatives pS, pT, and pY (bottom row). (**C**) Chemical structures of the phosphorylated amino acids phosphoserine, phosphothreonine, and phosphotyrosine (from left to right, the phosphate group is labeled in orange). (**D**) Aspartate (top) and glutamate (bottom) that are used as phosphomimetics. Both have a carboxylic group (red) with a charge of (−1) at physiological pH. (**E**) comparison between the spatial arrangement of the phosphate group (left), with a terahedral structure, and the carboxylic group (right), with a trigonal planar structure.

Each phosphate group introduces two negative charges into the protein at physiological pH (Figure 1A). The first pKa values of the phosphatidyl group are 2.8–3.2, thus not relevant for the native conditions in the cell. The second pKa values of the phosphorylated residues are 6.01 for phospho-serine (pS), 6.3 for phospho-threonine (pT), and 5.8 for phospho-tyrosine (pY) [9]. The negatively charged phosphate group is an excellent hydrogen-bond acceptor. It strengthens ionic interactions and forms salt bridges with side chains of lysine and arginine. Together, these interactions stabilize and rigidify local protein structures [10,11]. The excessive steric hindrance introduced by phosphorylation is an additional factor influencing protein stability, function, and interactions. The bulky phosphate group can affect protein structure by disrupting side-chain packing and backbone conformation, essentially pushing nearby residues into new positions. The steric hindrance also disrupts binding interfaces, thereby regulating the structural and functional behavior of proteins [12]. The side-chain volumes of pS and pT are approximately double that of the unmodified residues, while phosphorylated tyrosine exhibits an ∼1.5-fold increase in volume compared with its unmodified form (Figure 1B,C). Although Asp and Glu are often used as phosphomimetic substitutions because they introduce negative charge, they do not reproduce the steric and chemical properties of the phosphate group (Figure 1D,E). Finally, phosphate groups are strongly hydrophilic, tending to remain solvent-exposed and thereby shaping folding pathways and surface properties. By changing local stability, they can cause local unfolding, shift helix/strand tendencies, and switch between ordered and disordered states [13].

## Phosphorylation occurs mainly in intrinsically disordered regions

Phosphorylation is especially enriched in intrinsically disordered protein regions. Large-scale structural and proteomic surveys that project phosphosites onto predicted structures show that most modification events fall into disordered or low-structure domains rather than well-folded ones [14–19]. For example, analyses of human phosphoproteomes associated with biomolecular condensates found that the majority of phosphosites lie within predicted disordered segments, consistent with global mapping of human phosphosites that highlights a strong bias toward disordered or flexible regions [20]. Recent integrative work combining phosphoproteomics with AlphaFold-based structure prediction estimated that roughly 65% of all phosphorylation events occur in low-structured regions, in line with earlier observations that fully disordered proteins can harbor more than ten times as many phosphosites as fully ordered ones [21].

## Phosphorylation regulates protein function, localization, and interactions

**Figure 2:**
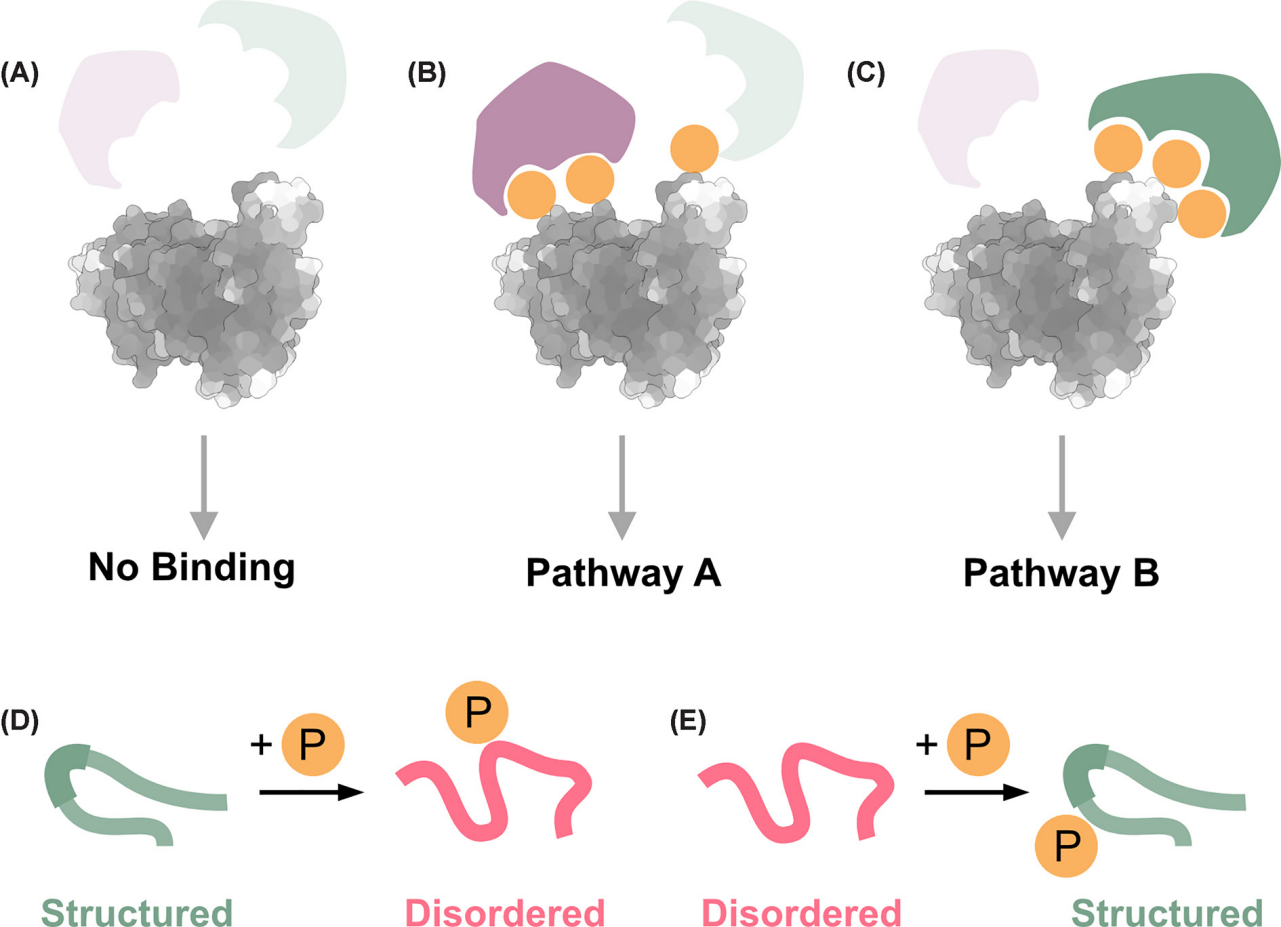
Distinct phosphorylation patterns modulate protein structure and interactions and lead to different biological pathways (**A**) An unphosphorylated protein (gray) with two potential binding partners (green and purple) that do not interact with it. (**B, C**) Specific phosphorylation patterns enable selective binding to the purple binding partner, activating pathway A, or to the green binding partner, activating pathway B. (**D, E**) Phosphorylation of proteins can transition from structured to disordered conformations (panel A) and *vice versa* (panel E).

Phosphorylation modulates a wide range of protein properties, including its function, subcellular localization, and molecular interactions (Figure 2A–C) and conformation (Figure 2D,E) [22–27]. The self-assembly of proteins can be either enhanced or suppressed by phosphorylation, leading to various oligomeric states of proteins, including soluble protein monomers, condensates, higher-order oligomers, amorphous aggregates, and amyloids [28–31]. Condensates form via liquid–liquid phase separation (LLPS) and can serve both as membrane-less organelles that catalyze chemical reactions [32–35] and as intermediates in aggregation processes [28–31]. Specific phosphorylation patterns do not only determine if a protein will self-associate but also control the self-association type. For example, phosphorylation regulates aggregation and condensation processes in neurodegeneration-related proteins [36–39]. In the Tau protein, hyperphosphorylation causes aggregation in neurodegenerative diseases. Tau phosphorylation regulates microtubule binding and can shift the protein toward LLPS or amyloid fibril formation in disease [32,40–47]. However, reproducing physiologically relevant Tau phosphorylation patterns *in vitro* remains challenging given the large number of sites modified *in vivo* [48–51].

Phosphorylation acts as a switch for conformational changes. Comparative structural analysis of phosphorylated and non-phosphorylated PDB structures of proteins such as GK, CLK1, MAP kinases, and PRP4K shows that phosphorylation induces modest structural changes, stabilizes local domains and modifies residue flexibility [52]. Phosphorylation also regulates protein–protein interactions (PPIs) by forming or disrupting binding interfaces. For instance, in the RAF pathway, phosphorylation of BRAF at Ser365 and Ser729 forms binding sites for a 14-3-3 dimer. 14-3-3 binding then stabilizes the BRAF–MEK1 complex in an inactive conformation that prevents cellular proliferation [53]. Similarly, phosphorylation regulates the interactions of the LRRK2 protein by forming binding sites for 14-3-3 proteins. In LRRK2, phosphorylation at Ser910 and Ser935 forms binding sites for a dimer of 14-3-3 proteins. 14-3-3 binding then stabilizes LRRK2 in an inactive conformation and inhibits its kinase activity [54]. Phosphorylation can also switch off an interaction as demonstrated for phosphorylation at Ser416 of the Tau protein, which inhibits binding to the E3 ligase CHIP [55].

Multisite phosphorylation can act combinatorially as distinct patterns function as molecular ‘barcodes’ that are interpreted by binding partners and regulatory machinery, shifting conformational ensembles and selectively changing PPI networks [56]. This was well-demonstrated for GPCR signaling, where different kinase-dependent phosphorylation patterns on receptor intracellular tails lead to distinct β-arrestin conformations and different downstream functional outcomes [4,57]. The intrinsically disordered C-terminal domain (CTD) of RNA polymerase II carries repeated heptads whose stage specific phosphorylation patterns orchestrate the sequential recruitment of transcription and RNA-processing factors across initiation, elongation, and termination [58,59].

## The need for systematic studies of the effects of phosphorylation patterns in proteins

**Figure 3:**
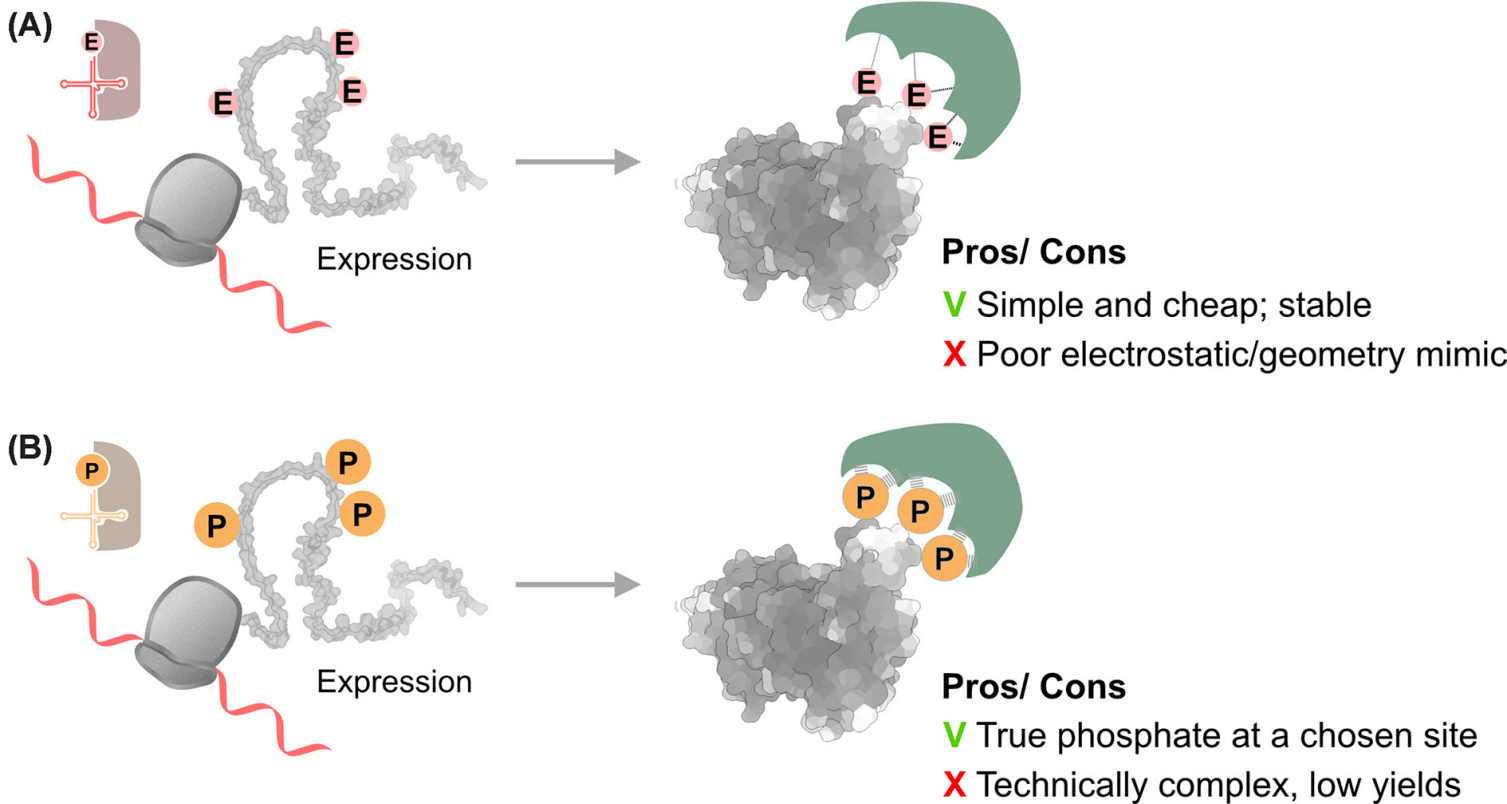
Strategies for studying phosphorylation patterns in cells (**A**) Phosphomimetics: expression of proteins bearing charge-mimicking substitutions (pS/pT/pY→E/D) to mimic the negative charge of the phosphate. RNA is shown as a red ribbon, glutamates as red circles, binding partners in green, and the interactions with black dotted lines. (**B**) Genetic code expansion: site-specific incorporation of phosphorylated residues using an orthogonal tRNA/aaRS system; tRNA depicted in orange.

Due to the complexity and context-dependence of phosphorylation effects, understanding their functional outcomes requires systematic study of defined phosphorylation patterns. This remains a major challenge due to the limitations of current methodologies for generating site-specific phosphorylated proteins. While cell-based assays are ideal for examining proteins in their native environment, it is difficult to systematically study phosphopatterns in the cellular environment. While studies at the cellular level are carried out under native conditions, they offer limited control over phosphorylation sites and stoichiometry, yielding mixed phosphorylation patterns that make it hard to link one site to a specific effect (Figure 3). Continuous kinase and phosphatase activity, together with spontaneous dephosphorylation, further reshapes these patterns over time, generating mixtures of multi-phosphorylated proteins that are difficult to analyze. *In vitro* studies, by contrast, allow isolation of defined sites and produce homogeneous phosphorylation patterns, enabling quantitative, mechanistic readouts of binding, stability, and conformational change. However, these reductionist *in vitro* systems do not operate under native cellular conditions and lack the full cellular environment that contains crowding agents, cofactors, and compartmentalization that modulate phosphorylation-dependent behavior. To bridge this gap, several strategies have been developed to generate multi-phosphorylated proteins or peptides. These strategies are complementary, and combining multiple approaches can yield a more comprehensive understanding of the biological system [13,60]. Below we describe the major methods used to study the effects of phosphorylation patterns in proteins in cells and *in vitro*, highlighting the pros and cons of each one of them and how they complement each other.

## Methods used in cells

### Phosphomimetics

The most common strategy for evaluating the effects of phosphorylation patterns in cells involves substituting the native Ser or Thr with a negatively charged glutamate to mimic phosphorylated residues (Figure 3A). These point mutations can be introduced either *in vitro* or in living cells [61,62]. Because they are genetically encoded, scalable, and compatible with cellular regulatory mechanisms, phosphomimetics are most likely to be informative when phosphorylation acts primarily by introducing negative charge or shifting local electrostatics (e.g., modulating intramolecular interactions). Practical considerations for selecting phosphomimetics are often site- and protein-dependent. For Ser/Thr sites, both Asp and Glu are commonly used. Glu provides a longer side chain while Asp is shorter, and either may better approximate the local electrostatic perturbation depending on the structural context. Thus, testing both can be informative when feasible. For Tyr phosphorylation, Tyr to Glu is generally a poor chemical mimic because it removes the aromatic ring and can alter recognition motifs. Accordingly, Tyr to Phe is typically used as a phospho-null control, while authentic pTyr is better addressed by orthogonal approaches. In the MAPK cascade, activation-loop phosphomimetic mutants have been used to generate constitutively active kinases for cellular studies. Constitutively active MAPKK constructs carrying activation-loop substitutions (S222D) are routinely used as pathway-activating tools in biochemical and cellular assays [63].

Phosphomimetic mutants, while having the advantage of the ability to work in the cellular environment, do not faithfully replicate the properties of native phosphoproteins. There is a fundamental chemical difference between a phosphate group and a carboxyl group. Replacing a pS or pT with glutamate partly mimics the negative charge but fails to reproduce the full chemical and structural characteristics of the phosphate group [61]. At physiological pH, a phosphate carries two negative charges, whereas glutamate carries only one. Their pKa differ substantially, with values of 5.58 for phosphate and 4.25 for glutamate [64]. Additionally, the phosphate group is more sterically hindered due to its tetrahedral geometry, unlike the smaller planar carboxylate (Figure 1C–E). These distinctions affect local interactions and conformational dynamics, influencing protein behavior at the site-specific level. Comparative studies have demonstrated that glutamate substitution does not always recapitulate the structural or functional outcomes of true phosphorylation [65]. For example, in α-synuclein, phosphorylation at Ser129 was shown to inhibit fibril formation, while S219E and S219D mutants failed to reproduce this effect [61,66].

Phosphomimics are useful initial tools, but since they provide only a partial and often inaccurate information, systematic studies of biologically relevant phosphorylation patterns remain highly challenging at the protein level. For example, in the 14-3-3 protein family, the canonical interaction is a direct binding between 14-3-3 and phosphorylated Ser/Thr motifs in their partner proteins. In this case, the phosphorylated motif is specifically recognized by the Arg–Arg–Tyr triad, which is a conserved basic pocket of 14-3-3. Asp/Glu do not fully mimic the geometry and charge distribution of the phosphate group and, thus, phosphomimetic substitutions do not actually serve as precise mimetics. For instance, BAD S136D/E mutants lose 14-3-3 binding, where phosphorylation supports strong association [10,61,67]. Following phosphorylation on Ser58 of 14-3-3, which is located at the dimer interface, phosphorylated 14-3-3 displays a change in its oligomeric state and protein localization, but the Glu mutant does not [56]. SH2 domains make another example demonstrating the limitations of the approach. SH2 domains bind pTyr using a conserved, positively charged pocket that makes key electrostatic interactions with the phosphate. In lipin-1 mutants in which three Src-targeted tyrosines were replaced by Asp, Glu, or Phe, the resulting proteins did not recapitulate the phosphorylation-dependent increase in lipin-1 activity, concluding that these substitutions cannot mimic Src-mediated tyrosine phosphorylation [68,69].

### Alanine scan

Alanine scan is a common strategy to determine the importance of specific phosphorylations. In this approach, Ser/Thr are substituted with Ala, and Tyr is substituted with Phe to prevent phosphorylation at specific locations in cells by preventing kinases from phosphorylating these residues [70]. A key advantage of these mutants is that they are straightforward to implement in cells and tissues, enabling functional tests under physiological conditions. Alanine scan was used to obtain all possible 256 mutants of the yeast transcription factor Hcm1, which has eight CDK phosphorylation sites. This strategy showed that distinct patterns can retain near-wild-type Hcm1 function *in vivo* and pinpointed T460/S471 as important contributors [70,71].

The downside of this method is that it provides information on the requirement for phosphorylation at specific sites but does not indicate whether this is required for a specific function. Replacing a Ser/Thr hydroxyl with the methyl group of alanine prevents the formation of potential hydrogen bonds, which might disrupt local interactions and structure, rather than just prevent phosphorylation [72,73]. Tyr to Phe substitution is more conservative than Ser/Thr to Ala because it preserves the aromaticity. Yet it still removes a polar hydroxyl that may contribute to binding or catalysis. Alanine scan results are therefore best interpreted alongside complementary approaches to distinguish loss of phosphorylation from mutation-induced structural or interaction effects.

### Genetic code expansion

Another method for evaluating the function and role of specific phosphorylation patterns is genetic code expansion (Figure 3B). This method enables the incorporation of unnatural amino acids into proteins during ribosomal translation. It uses an unassigned codon, a matching tRNA, and a corresponding aminoacyl-tRNA synthetase that specifically charges the tRNA with the desired unnatural amino acid, all functioning orthogonally to the endogenous translation machinery of the host [74–77]. Systems for genetic code expansion have been developed in cell-free, bacterial, and animal models, providing a powerful approach for studying phosphorylated proteins in cells [78]. Using this strategy, researchers have successfully introduced pS, pT and pY into target proteins [79–82]. Engineered strains and plasmid toolkits for the incorporation of phosphorylated amino acids into recombinant proteins during expression and purification are now widely available. The use of these requires an orthogonal translation system, as well as optimization of suppression efficiency and expression conditions. For example, as a proof-of-principle that genetic code expansion can be scaled in bacteria, a recoded *Escherichia coli* host with a phosphoserine orthogonal translation system was used to express a peptide library with more than 100,000 members containing pSer. This enabled proteome-wide screens for phosphorylation-dependent interactions [83]. Despite its advantages, the method faces several challenges. Competition with release factors at stop codons often leads to low expression levels. Furthermore, mischarging of tRNAs may result in the incorporation of natural amino acids, compromising fidelity. The limited pool of unassigned codons also limits the number of unnatural residues that can be incorporated simultaneously. As a result, this approach may produce heterogeneous mixtures of phosphoproteins rather than a single, well-defined phosphorylation pattern.

## *In vitro* methods

### *In vitro* enzymatic phosphorylation

**Figure 4:**
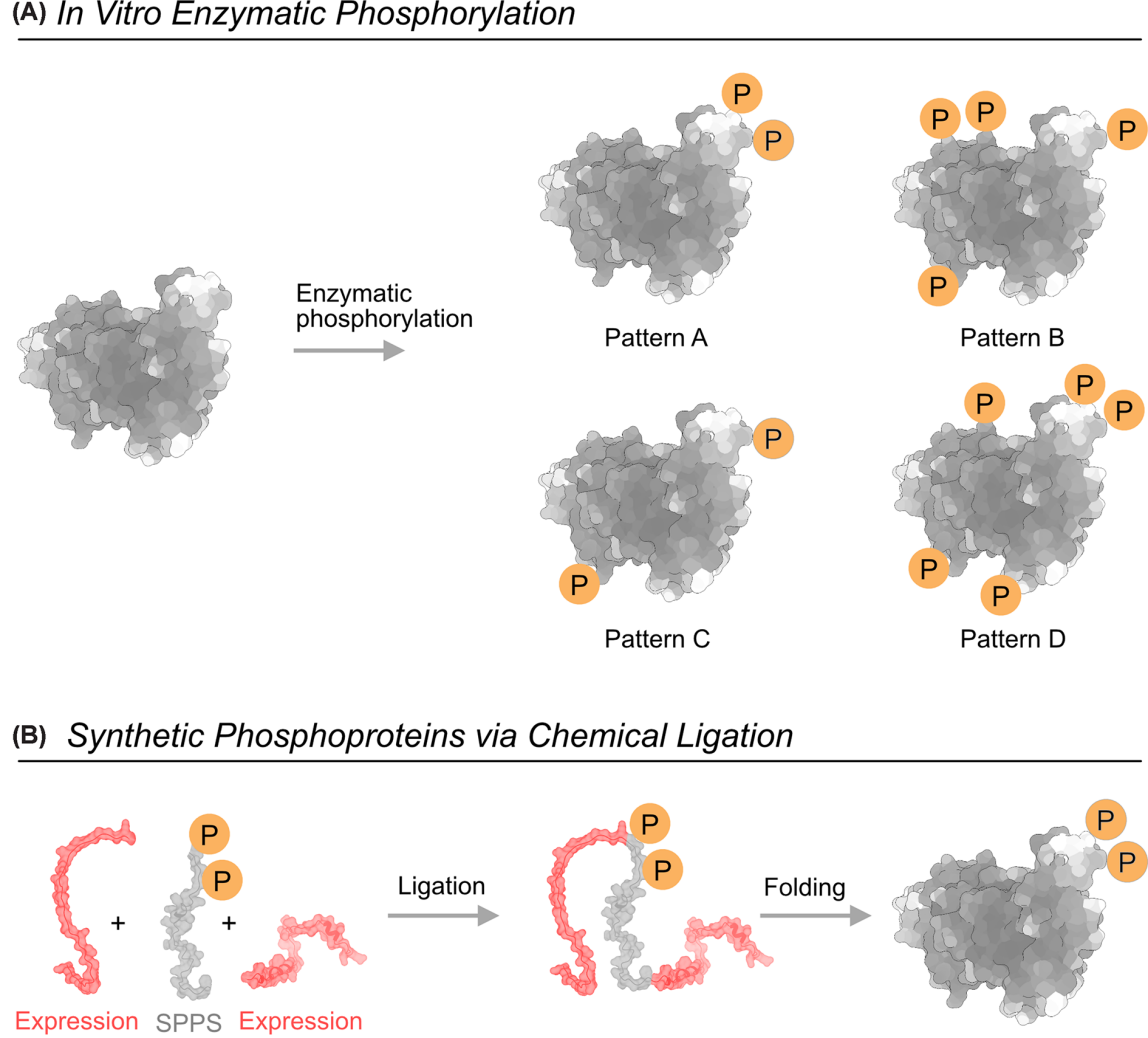
Synthetic approach for obtaining phosphoproteins (**A**) *In vitro* enzymatic phosphorylation. (**B**) Chemical ligation approach. Phosphorylation sites are labeled in orange circles. For ligation, the expressed protein is labeled in red.

Enzymatic phosphorylation is widely used for generating phosphoproteins *in vitro* and is particularly effective for exploring links between phosphorylation and protein function [82]. While kinases can produce native phosphorylation patterns, this approach comes with several limitations (Figure 4A). It depends on prior knowledge of which kinase modifies each target site, information that is often incomplete or unknown [84]. Furthermore, many kinases exhibit limited specificity, leading to the formation of heterogeneous mixtures containing multiple phosphorylation patterns that can be difficult to resolve [85]. Collectively, these limitations may result in under-phosphorylation of key sites due to inefficient kinase activity and/or over-phosphorylation at some sites (Figure 4A).

### Synthetic phosphoproteins produced via chemical ligation

Chemical ligation techniques, such as native chemical ligation (NCL), offer a robust platform for generating phosphoproteins with high precision [86–91]. NCL is used to link a peptide bearing a C-terminal thioester to a second peptide with an N-terminal cysteine, forming a native peptide bond [91–94]. By incorporating one or more phosphorylated residues into the peptide segments, this strategy can be applied to the synthesis of phosphorylated proteins. These methods enable the site-specific incorporation of phosphorylated residues and other PTMs that are often inaccessible through recombinant expression. Approaches like NCL and Ser/Thr ligation allow the modular assembly of full-length proteins from smaller peptide segments, overcoming the size limitations inherent to solid-phase peptide synthesis (SPPS) [13,95]. A key advantage of these strategies is the ability to generate well-defined phosphorylation patterns, which is critical for dissecting the specific biological roles of individual phosphorylation patterns. However, chemical ligation is not without significant constraints. It depends heavily on the successful synthesis of phosphorylated peptides in sufficient yield and purity. In practice, the success of NCL-based phosphoprotein synthesis is tightly coupled to the availability of multi-phosphorylated peptide (MPP) fragments that are ‘NCL-ready’. The challenges associated with preparing such fragments will be discussed later in this review, in the ‘Chemical strategies for multi-phosphorylated peptide synthesis’ section. NCL typically requires a cysteine residue at the ligation site, which can necessitate unnatural sequence modifications and additional desulfurization steps. The preparation and manipulation of peptide thioesters also demand specialized handling, especially when labile phosphate groups are involved [96,97]. Alternative ligation approaches also exist. Auxiliary-based ligation uses a removable thiol-containing auxiliary to enable NCL-like coupling at non-cysteine junctions, followed by auxiliary cleavage to regenerate the native residue [98,99]. KAHA chemistry enables native amide-bond formation under mild aqueous conditions and can be advantageous for PTM-rich targets [100,101].

Enzyme-mediated ligation, including intein-assisted protein splicing and sortase-catalyzed transpeptidation, provides highly site-selective assembly in water and can complement chemical ligations for larger or more complex constructs [102,103]. These methods are used to ligate a synthetic phosphopeptide segment to a recombinant protein, resulting in a single, well-defined phosphoform. Several enzyme families can be used for this purpose. For example, sortase-catalyzed ligation was used to attach a phosphorylated GPCR C-terminal peptide to purified receptors, enabling controlled studies of how tail phosphorylation patterns tune coupling of β-arrestin [104]. Intein-mediated expressed protein ligation was used to ligate synthetic phosphopeptides to intein-generated protein thioesters and produce arrayed phosphoprotein substrates for kinase/phosphatase assays and phospho-dependent screening [105]. These approaches expand the range of compatible sequences by overcoming some key constraints of NCL: auxiliaries enable coupling at non-cysteine junctions, KAHA avoids the need for peptide thioesters, and intein or sortase systems use short recognition elements to achieve ligation in sequence contexts that are otherwise difficult to access chemically. However, these methods also come with trade-offs like low efficiency, harsh reaction conditions, or added synthetic complexity [102,106–111]. Despite the versatility and precision, both chemical and enzymatic ligation approaches require unique expertise. The preparation of each synthetic phosphoprotein requires extensive optimization and yields only small quantities (Figure 4B). This makes this labor-intensive and difficult-to-scale technique available only to specialized labs.

Overall, when kinase reactions yield heterogeneous mixtures or miss low-efficiency sites, protein semisynthesis by enzymatic ligation offers a direct way to install a defined phospho-segment with fixed stoichiometry.

## Studies at the domain level using synthetic phosphopeptides

A common way for avoiding the problems associated with phosphoproteins is to work at the domain level. Synthetic phosphorylated peptides (phosphopeptides), often representing specific protein domains, offer a precise and versatile platform for investigating the functional impact of phosphorylation. This is applicable for small protein domains that can be chemically synthesized. Phosphopeptides allow exact control over the number and position of phosphosites, making them valuable for mechanistic studies and for guiding the development of kinase inhibitors [4,112–114]. This comes with the price of not working at the protein level, making this method complementary to the other ones mentioned above. In many cases phosphosites are located within intrinsically disordered regions (see the ‘Phosphorylation occurs mainly in intrinsically disordered regions’ section above), so short phosphopeptides may faithfully capture the local, disordered environment of the modification sites without disrupting a pre-existing folded core. In such systems, phosphopeptide libraries provide a structurally meaningful and experimentally accessible surrogate that can be used to screen phosphorylation patterns and then focus time- and resource-intensive protein-level methods on a small number of functionally relevant phosphorylation patterns identified at the peptide level.

The term ‘library’ describes a variety of possible scales in peptide research, including phosphopeptide research. This scale can range from a single-digit collection of different peptides to very big combinatorial libraries of >100,000 peptides [115]. For example, a library of 25,196 sequences was designed for PP1/PP2A substrate profiling [116]. In many other cases, small libraries of <10 peptides were sufficient to generate valuable information. For example, 5/6 RNAPII CTD diheptad phosphopeptides [117,118], an eight-member library, were used to compare 14-3-3 isoform binding mechanisms [119], a seven-peptide SRM library to quantify site-specific phosphorylation [120], or a four-member library that contains all phosphorylation patterns of phospholamban [121].

Of course, the context of the full protein cannot be overlooked, and in many other cases, especially when the disordered regions interact with structured domains, the picture is more complex, and structural effects have to be taken into account. This is the case when phosphorylation sites within disordered regions modulate an enzyme or transporter. A main challenge when using this method is the synthesis of MPPs, as steric hindrance, electrostatic repulsion, and β-elimination lower the efficiency of the synthesis. Several synthetic strategies were developed by us and others to solve these problems and allow the efficient synthesis of MPP libraries.

## Chemical strategies for multi-phosphorylated peptide synthesis

Two principal chemical strategies are used for obtaining MPPs: the global phosphorylation approach and the building-block (BB) approach. In the global phosphorylation approach, the phosphate groups are introduced chemically after the peptide has been fully assembled. Using phosphoramidate reagents, phosphorylation proceeds via two steps: phosphitylation of hydroxyl moieties followed by oxidation [122–125]. This method, reviewed in detail in [60], is less popular nowadays, as it is laborsome, time-consuming, and requires special expertise. The BB approach is the most commonly used method for synthesizing phosphopeptides [13,60]. It involves the incorporation of protected phosphorylated amino acids (such as monobenzyl-protected pS and pT) into peptides via Fmoc-based SPPS [126,127]. While effective, this strategy becomes increasingly complex when trying to synthesize peptides with more than three phosphorylated residues, particularly when they are clustered. The bulky phosphate groups decrease coupling efficiency and are prone to β-elimination under basic and heated conditions, especially during Fmoc deprotection [126,128]. Since elimination and Fmoc deprotection occur under the same conditions, careful control of temperature and pH is required.

BB-based strategies for MPP synthesis can be performed using automated or manual microwave-assisted peptide synthesis [129]. Peptides bearing up to three phosphorylation sites can be synthesized using the standard automated SPPS methods. However, the synthesis of MPPs with clustered phosphorylated regions or/and a higher number of phosphorylations is very difficult. The synthesis of a small library of heptad-repeat peptides derived from the C-terminal domain of RNA polymerase II with multiple phosphorylation sites was demonstrated using a BB strategy [86]. Although successful, this protocol involved long coupling and deprotection steps and large excesses of phosphorylated building blocks. Using a flow-chemistry setup, a highly phosphorylated peptide was prepared using a very large BB excess and still resulted in a low isolated yield [130]. Together, these examples illustrate that each synthetic method carries characteristic trade-offs in scale, practicality, accessibility, and speed, underscoring the value of accelerated MPP synthesis protocols that can deliver comparable or superior targets with markedly reduced synthetic effort.

To obtain libraries of MPPs, we have used microwave-assisted peptide synthesis with specifically optimized conditions for each peptide. This method was used to study the effect of multi-phosphorylation on the interactions of the C-terminal tail of rhodopsin [4,131]. While being excellent for making specific MPPs, this method was still manual and time-consuming. For automating the synthesis, we used the Glyconeer 2.1 carbohydrate synthesizer that has the ability not only to heat but also to cool. This was combined with extended coupling and deprotection steps [132], resulting in an automated protocol that decreased the synthesis times from weeks to days while maintaining high crude purity for long, clustered multi-phosphorylated sequences. The Glyconeer 2.1 thus provides an ideal, but specialized, platform for automated MPP synthesis, and its use will naturally be limited to laboratories that have access to this instrument. A second, accelerated protocol was then established, based on constant high temperature, vigorous stirring, and optimized Fmoc deprotection conditions, replacing the piperidine base, which is routinely used, with DBU [133]. This high-diffusion chemistry shortened the synthesis to minutes and yielded milligram quantities of MPPs [133–136].This accelerated protocol, in contrast with the specialized Glyconeer-based protocol, can be implemented in any synthetic laboratory. It requires only a heated, stirred reactor and commonly available laboratory equipment.

## Comparison between the common methods for studying phosphorylation pattens in proteins

In comparing the available approaches, three criteria are particularly important: (i) whether the protein is studied in a native cellular context or *in vitro*; (ii) the type of readout each method best supports, from cellular and functional assays to quantitative *in vitro* binding, kinetic, and structural measurements; and (iii) its practical accessibility in terms of yields, time, and the ability to generate libraries of phosphorylation patterns. In the context of multi-phosphorylation, the number of possible phosphorylation patterns increases combinatorially with the number of sites, so studying individual examples in isolation is rarely sufficient. Libraries of defined phosphorylation patterns enable systematic, side-by-side comparison of many patterns under identical conditions, allowing unbiased identification of phosphorylation patterns that promote or inhibit molecular events such as binding, structural changes, condensation, or aggregation that ultimately lead to different cellular pathways. In general, approaches that are closer to the native cellular environment tend to be more biologically relevant but less controlled, whereas *in vitro* and peptide-based approaches sacrifice some physiological context in favor of mechanistic precision and throughput.

Table 1 summarizes the advantages and disadvantages of each method, showing their complementarity. Ideally, it is always best to study phosphorylation patterns in proteins using more than one method. Kinase-based phosphorylation approach allows studying the protein in its native regulatory environment. The limitation is heterogeneity and variable stoichiometry, which often necessitate repeated enrichment steps. Consequently, the practical isolated yield of a single, well-defined phosphoform is decreased, and the time to product is extended. Phosphomimetic proteins answer a narrower question: does added negative charge at a given site result in a specific function, localization, or choice of an interaction partner? The method is used for rapid in-cell screens and can be coupled to basic biochemical assays [20,70,137]. Because they are not mimicking the full properties of phosphates regarding charge, size, geometry, and mechanistic, structural conclusions are commonly inaccurate and must be validated against true phosphorylation patterns.

**Table 1:** Comparison between the common methods for studying phosphorylation patterns in proteins*

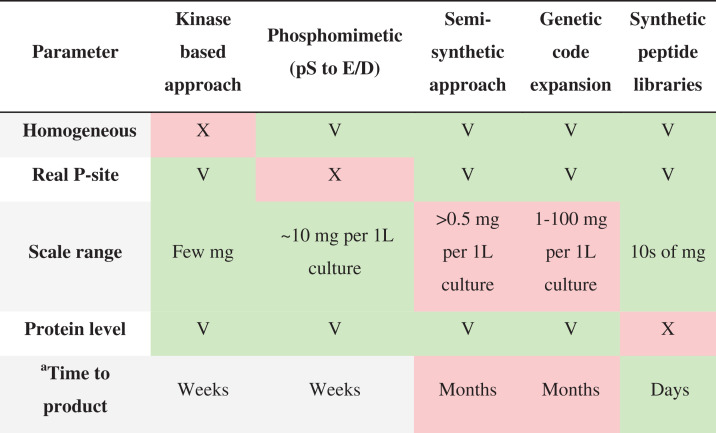	

*‘V’ indicates that the method reliably supports the given parameter. ‘X’ means the method does not support or achieve that parameter. Green-shaded box highlights the main advantages associated with each method. Red-shaded boxes highlight the main limitations or shortcomings associated with each method. ^a^ Time denotes the end-to-end time to a purified, analytically characterized product.

Semi-synthetic phosphoproteins provide full-length, authentic, homogeneous constructs and therefore allow a definitive, site-resolved mechanism study. However, they enable limited quantitative biophysical studies (binding, kinetics, stability, conformation/dynamics) due to the small amount of proteins produced, usually less than 0.5 mg per 1 L culture. The cost in time, specialized chemistry, a multi-step protocol, and cumulative losses during ligation make it difficult to produce libraries using this method. The synthetic phosphopeptide approach is the only one that allows obtaining high quantities of homogeneous phosphorylation patterns. The yields range from a few milligrams to hundreds of milligrams, depending on the specific sequence. This enables a wide spectrum of biophysical and biochemical studies. However, working with phosphopeptides requires further examination at the protein level.

Genetic code expansion provides a complementary route, enabling in-cell expression of full-length proteins bearing site-specifically incorporated phosphoserine or phosphate-mimicking non-canonical amino acids at defined positions. This approach allows direct analysis of individual phosphorylation events in a native cellular context using standard cell-based assays. Its main limitations are the need for engineered expression systems, practical constraints on the number and positions of sites, and the requirement for specialized expertise and infrastructure that are currently available only in a limited number of laboratories. Reported yields are usually low (mg/L) but can vary to high mg/l for optimized single-site incorporation [138]. Taken together, these methods span the spectrum from highly biologically relevant but less controlled (kinase-based, phosphomimics, and genetic code expansion) to highly controlled and library-friendly but less native (synthetic phosphopeptides and, to a lesser extent, semi-synthetic phosphoproteins), as summarized in Table 1.

## Practical considerations

Functional interrogation of phosphorylation sites in cells and tissues most often starts with non-phosphorylated mutants such as Ser/Thr to Ala or Tyr to Phe, often complemented by phosphomimetics. These approaches are experimentally accessible and scalable, but they do not provide homogeneous, stoichiometrically defined phosphoforms. Functional studies in cells and tissues commonly begin with genetically encoded site substitutions coupled to cellular phenotyping. Mechanistic dissection, particularly for multisite phosphorylation patterns, often requires follow-up studies using approaches that generate defined phosphoforms and/or combinatorial mapping strategies to prioritize key site combinations for protein-level validation.

## Case study: domain-level phosphopeptides for understanding Tau self-assembly

We used domain-level synthetic phosphopeptides to explore the specific effects of phosphorylation patterns on the self-assembly of the Tau R4 domain. Tau represents an extremely complex case due to extensive hyperphosphorylation at multiple sites. To manage this complexity, in addition to full-length protein studies, Tau has been extensively investigated at the domain level. One notable example is the resolution of the first cryo-EM structure of an intermediate filamentous Tau species (FIA), which was achieved using a 97-residue fragment of Tau. This highlighted the importance of focusing on structurally relevant regions [85]. Another widely used model is the K18 fragment, which includes the microtubule-binding repeats of Tau and has been a reliable system for studying its mechanism of aggregation [86–88]. Short peptides are also excellent models for structural and mechanistic studies of Tau. The short hexapeptides PHF (Tau 306–311) and PHF* (Tau 275–280) from the microtubule-binding region of Tau are widely used as minimal models to study Tau aggregation. These peptides, which share a canonical motif of V-Q-I-ϕ-x-K, where ϕ stands for an aliphatic residue, are the major aggregation hotspots within Tau, and their self-assembly into β-sheet-rich structures underlies the nucleation of amyloid fibrils in Alzheimer's disease and related tauopathies [139,140].

We have produced a synthetic library of phosphorylated peptides derived from the R4 domain, the fourth repeat of the microtubule-binding region of Tau, to systematically assess the impact of specific phosphorylation patterns on Tau self-assembly [141]. By working at the domain level with fully synthetic, homogeneously phosphorylated R4 peptides, we could isolate the contribution of the phosphorylation pattern itself, decoupling it from other Tau regions, binding partners, and cellular cofactors, and directly compare its effects on condensation and aggregation under identical solution conditions. Eight distinct phosphorylation patterns were synthesized, covering all possible combinations of phosphorylation at three sites: Ser341, Ser352, and Ser356. The results revealed that phosphorylation at Ser352 promotes condensation, Ser341 promotes aggregation, and phosphorylation of Ser356 suppresses both processes. These findings demonstrate that individual phosphorylation sites, and more importantly, their combinations, act as molecular switches that modulate the transition of Tau protein between functional condensation and pathological aggregation [141]. In this focused example, a small, well-defined library comprising all combinations of three phosphorylation sites was sufficient to distinguish phosphorylation-dependent condensate formation from aggregation in Tau R4.

## Summary and conclusions

The methods described above are complementary and provide different viewpoints for phosphorylation studies. Kinase-based and phosphomimetic strategies are ideal for rapid cell-level screening and hypothesis generation. Semi-synthetic approaches provide definitive, full-length phosphorylation patterns to resolve targeted mechanistic questions at the protein level. Synthetic phosphopeptide libraries enable scalable mapping of site combinations, order effects, and local mechanisms. It is a starting point for selecting with which phosphorylation pattern one should work at the protein level, particularly for intrinsically disordered, heavily phosphorylated proteins where domain-level phosphopeptides provide a structurally relevant guide for deciding which full-length constructs are worth preparing by the more demanding semi-synthetic or genetic-code-expansion approaches. As summarized in Table 1, each method carries distinct strengths that can be integrated into a coherent pipeline from discovery to mechanism. The complementary methods, each contributing unique advantages and addressing specific limitations, offer an integrated framework for systematically probing the complex regulation imposed by phosphorylation in biological systems.

Although this review focuses on phosphorylation, many of the concepts and methodologies developed for synthetic phosphopeptide libraries are readily extendable to other post-translational modifications, such as acetylation, methylation, and glycosylation. Applying analogous library-based strategies to these PTMs, and ultimately to combinations of modifications on the same peptide or domain, will enable systematic dissection of PTM crosstalk and broaden the toolbox for mapping how complex modification patterns regulate protein structure, interactions, and self-assembly.

## Perspectives

- Protein multiphosphorylation creates many possible patterns, where each pattern can drive a distinct functional or pathological outcome.
- Numerous methods exist to study the effects of multiphosphorylation patterns in proteins, but no single method can reliably generate protein derivatives with all relevant phosphorylation patterns. Progress depends on combining cellular, enzymatic, semi-synthetic, and synthetic strategies.
- Future directions will likely use integrated workflows in which scalable synthetic phosphopeptide libraries map domain-level pattern effects and guide focused protein-level validation, with the same logic expanding to other PTMs and combinations thereof.

## Open Access

Open access for this article was enabled by the participation of the Hebrew University of Jerusalem in an all-inclusive Read & Publish agreement with Portland Press and the Biochemical Society under an agreement with MALMAD.

## Abbreviations

BB: building-block
CTD: C-terminal domain
LLPS: liquid–liquid phase separation
MPP: multi-phosphorylated peptide
pS: phospho-serine
pT: phospho-threonine
pY: phospho-tyrosine
PTMs: post-translational modifications
PPIs: protein–protein interactions

## References

[1] Vieitez C., Busby B.P., Ochoa D., Mateus A., Memon D., Galardini M. et al. (2022) High-throughput functional characterization of protein phosphorylation sites in yeast. Nat. Biotechnol. 40, 382–390 10.1038/s41587-021-01051-x 34663920 PMC7612524

[2] Ubersax J.A. and Ferrell J.E. Jr (2007) Mechanisms of specificity in protein phosphorylation. Nat. Rev. Mol. Cell Biol. 8, 530–541 10.1038/nrm2203 17585314

[3] Paradela A. and Albar J.P. (2008) Advances in the analysis of protein phosphorylation. J. Proteome Res. 7, 1809–1818 10.1021/pr7006544 18327898

[4] Mayer D., Damberger F.F., Samarasimhareddy M., Feldmueller M., Vuckovic Z., Flock T. et al. (2019) Distinct G protein-coupled receptor phosphorylation motifs modulate arrestin affinity and activation and global conformation. Nat. Commun. 10, 1261 10.1038/s41467-019-09204-y 30890705 PMC6424980

[5] Palmeri A., Ausiello G., Ferre F., Helmer-Citterich M. and Gherardini P.F. (2014) A proteome-wide domain-centric perspective on protein phosphorylation. Mol. Cell. Proteomics 13, 2198–2212 10.1074/mcp.M114.039990 24830415 PMC4159644

[6] Narasimha A.M., Kaulich M., Shapiro G.S., Choi Y.J., Sicinski P. and Dowdy S.F. (2014) Cyclin D activates the Rb tumor suppressor by mono-phosphorylation. eLife 3, 10.7554/eLife.02872 24876129 PMC4076869

[7] Watamura N., Foiani M.S., Bez S., Bourdenx M., Santambrogio A., Frodsham C. et al. (2025) *In vivo* hyperphosphorylation of tau is associated with synaptic loss and behavioral abnormalities in the absence of tau seeds. Nat. Neurosci. 28, 293–307 10.1038/s41593-024-01829-7 39719507 PMC11802456

[8] Kimura T., Sharma G., Ishiguro K. and Hisanaga S.I. (2018) Phospho-tau bar code: analysis of phosphoisotypes of tau and its application to tauopathy. Front. Neurosci. 12, 44 10.3389/fnins.2018.00044 29467609 PMC5808175

[9] Hendus-Altenburger R., Fernandes C.B., Bugge K., Kunze M.B.A., Boomsma W. and Kragelund B.B. (2019) Random coil chemical shifts for serine, threonine and tyrosine phosphorylation over a broad pH range. J. Biomol. NMR 73, 713–725 10.1007/s10858-019-00283-z 31598803 PMC6875518

[10] Yang X., Lee W.H., Sobott F., Papagrigoriou E., Robinson C.V., Grossmann J.G. et al. (2006) Structural basis for protein–protein interactions in the 14-3-3 protein family. Proc. Natl. Acad. Sci. U.S.A. 103, 17237–17242 10.1073/pnas.0605779103 17085597 PMC1859916

[11] Zhang W., Chen Y., Guan Z., Wang Y., Tang M., Du Z. et al. (2025) Structural insights into the mechanism of phosphate recognition and transport by XPR1. Nat. Commun. 16, 10.1038/s41467-024-55471-9 PMC11696373 39747008

[12] Pandey A.K., Ganguly H.K., Sinha S.K., Daniels K.E., Yap G.P.A., Patel S. et al. (2023) An inherent difference between serine and threonine phosphorylation: phosphothreonine strongly prefers a highly ordered, compact, cyclic conformation. ACS Chem. Biol. 18, 1938–1958 10.1021/acschembio.3c00068 37595155

[13] Bilbrough T., Piemontese E. and Seitz O. (2022) Dissecting the role of protein phosphorylation: a chemical biology toolbox. Chem. Soc. Rev. 51, 5691–5730 10.1039/D1CS00991E 35726784

[14] Newcombe E.A., Delaforge E., Hartmann-Petersen R., Skriver K. and Kragelund B.B. (2022) How phosphorylation impacts intrinsically disordered proteins and their function. Essays Biochem. 66, 901–913 10.1042/EBC20220060 36350035 PMC9760426

[15] Bludau I., Willems S., Zeng W.F., Strauss M.T., Hansen F.M., Tanzer M.C. et al. (2022) The structural context of posttranslational modifications at a proteome-wide scale. PLoS Biol. 20, e3001636 10.1371/journal.pbio.3001636 35576205 PMC9135334

[16] Holehouse A.S. and Kragelund B.B. (2024) The molecular basis for cellular function of intrinsically disordered protein regions. Nat. Rev. Mol. Cell Biol. 25, 187–211 10.1038/s41580-023-00673-0 37957331 PMC11459374

[17] Iakoucheva L.M., Radivojac P., Brown C.J., O'Connor T.R., Sikes J.G., Obradovic Z. et al. (2004) The importance of intrinsic disorder for protein phosphorylation. Nucleic Acids Res. 32, 1037–1049 10.1093/nar/gkh253 14960716 PMC373391

[18] Collins M.O., Yu L., Campuzano I., Grant S.G. and Choudhary J.S. (2008) Phosphoproteomic analysis of the mouse brain cytosol reveals a predominance of protein phosphorylation in regions of intrinsic sequence disorder. Mol. Cell. Proteomics 7, 1331–1348 10.1074/mcp.M700564-MCP200 18388127

[19] Holt L.J., Tuch B.B., Villén J., Johnson A.D., Gygi S.P. and Morgan D.O. (2009) Global analysis of Cdk1 substrate phosphorylation sites provides insights into evolution. Science 325, 1682–1686 10.1126/science.1172867 19779198 PMC2813701

[20] Sridharan S., Hernandez-Armendariz A., Kurzawa N., Potel C.M., Memon D., Beltrao P. et al. (2022) Systematic discovery of biomolecular condensate-specific protein phosphorylation. Nat. Chem. Biol. 18, 1104–1114 10.1038/s41589-022-01062-y 35864335 PMC9512703

[21] Bickel D. and Vranken W. (2024) Effects of phosphorylation on protein backbone dynamics and conformational preferences. J. Chem. Theory Comput. 20, 4998–5011 10.1021/acs.jctc.4c00206 38830621 PMC11210476

[22] Ardito F., Giuliani M., Perrone D., Troiano G. and Lo Muzio L. (2017) The crucial role of protein phosphorylation in cell signaling and its use as targeted therapy (Review). Int. J. Mol. Med. 40, 271–280 10.3892/ijmm.2017.3036 28656226 PMC5500920

[23] Cohen P. (2000) The regulation of protein function by multisite phosphorylation—a 25 year update. Trends Biochem. Sci 25, 596–601 10.1016/S0968-0004(00)01712-6 11116185

[24] Cohen P. (2002) The origins of protein phosphorylation. Nat. Cell Biol. 4, E127–E130 10.1038/ncb0502-e127 11988757

[25] Cohen P. (2001) The role of protein phosphorylation in human health and disease. Eur. J. Biochem. 268, 5001–5010 10.1046/j.0014-2956.2001.02473.x 11589691

[26] Day E.K., Sosale N.G. and Lazzara M.J. (2016) Cell signaling regulation by protein phosphorylation: a multivariate, heterogeneous, and context-dependent process. Curr. Opin. Biotechnol. 40, 185–192 10.1016/j.copbio.2016.06.005 27393828 PMC4975652

[27] Nishi H., Hashimoto K. and Panchenko Anna R. (2011) Phosphorylation in protein-protein binding: effect on stability and function. Structure 19, 1807–1815 10.1016/j.str.2011.09.021 22153503 PMC3240861

[28] Kumar C.M.S., Khare G., Srikanth C.V., Tyagi Anil K., Sardesai Abhijit A. and Mande Shekhar C. (2009) Facilitated oligomerization of mycobacterial GroEL: evidence for phosphorylation-mediated oligomerization. J. Bacteriol. 191, 6525–6538 10.1128/JB.00652-09 19717599 PMC2795288

[29] Schwarz J.K., Lovly C.M. and Piwnica-Worms H. (2003) Regulation of the Chk2 protein kinase by oligomerization-mediated *cis*- and *trans*-phosphorylation1. Mol. Cancer Res. 1, 598–609 12805407

[30] Carlomagno Y., Chung D.E.C., Yue M., Castanedes-Casey M., Madden B.J., Dunmore J. et al. (2017) An acetylation–phosphorylation switch that regulates tau aggregation propensity and function. J. Biol. Chem. 292, 15277–15286 10.1074/jbc.M117.794602 28760828 PMC5602388

[31] Kimchi O., Goodrich C.P., Courbet A., Curatolo A.I., Woodall N.B., Baker D. et al. (2020) Self-assembly–based posttranslational protein oscillators. Sci. Adv. 6, eabc1939 10.1126/sciadv.abc1939 33328225 PMC7744077

[32] Ambadipudi S., Biernat J., Riedel D., Mandelkow E. and Zweckstetter M. (2017) Liquid-liquid phase separation of the microtubule-binding repeats of the Alzheimer-related protein Tau. Nat. Commun. 8, 275 10.1038/s41467-017-00480-0 28819146 PMC5561136

[33] Jo Y. and Jung Y. (2019) Interplay between intrinsically disordered proteins inside membraneless protein liquid droplets. Chem. Sci. 11, 1269–1275 10.1039/C9SC03191J 34123251 PMC8148370

[34] Yewdall N.A., André A.A.M., Lu T. and Spruijt E. (2021) Coacervates as models of membraneless organelles. Curr. Opin. Colloid Interface Sci. 52, 101416 10.1016/j.cocis.2020.101416

[35] Astoricchio E., Alfano C., Rajendran L., Temussi P.A. and Pastore A. (2020) The wide world of coacervates: from the sea to neurodegeneration. Trends Biochem. Sci 45, 706–717 10.1016/j.tibs.2020.04.006 32417131

[36] Ansaloni A., Wang Z.M., Jeong J.S., Ruggeri F.S., Dietler G. and Lashuel H.A. (2014) One‐pot semisynthesis of Exon 1 of the Huntingtin protein: new tools for elucidating the role of posttranslational modifications in the pathogenesis of Huntington’s disease. Angew. Chem. Int. Ed. Engl. 53, 1928–1933 10.1002/anie.201307510 24446188

[37] Pan B., Rhoades E. and Petersson E.J. (2020) Chemoenzymatic semisynthesis of phosphorylated α-synuclein enables identification of a bidirectional effect on fibril formation. ACS Chem. Biol. 15, 640–645 10.1021/acschembio.9b01038 32065743 PMC7724256

[38] Monahan Z., Ryan V.H., Janke A.M., Burke K.A., Rhoads S.N., Zerze G.H. et al. (2017) Phosphorylation of the FUS low-complexity domain disrupts phase separation, aggregation, and toxicity. EMBO J. 36, 2951–2967 10.15252/embj.201696394 28790177 PMC5641905

[39] Tsang B., Arsenault J., Vernon R.M., Lin H., Sonenberg N., Wang L.-Y. et al. (2019) Phosphoregulated FMRP phase separation models activity-dependent translation through bidirectional control of mRNA granule formation. Proc. Natl. Acad. Sci. U.S.A. 116, 4218–4227 10.1073/pnas.1814385116 30765518 PMC6410804

[40] Pîrşcoveanu D.F.V., Pirici I., Tudorică V., Bălşeanu T.-A., Albu V.-C., Bondari S. et al. (2017) Tau protein in neurodegenerative diseases-a review. Rom. J. Morphol. Embryol. 58, 1141–1150 29556602

[41] Avila J., Lucas J.J., Perez M. and Hernandez F. (2004) Role of tau protein in both physiological and pathological conditions. Physiol. Rev. 84, 361–384 10.1152/physrev.00024.2003 15044677

[42] Buee L., Bussiere T., Buee-Scherrer V., Delacourte A. and Hof P.R. (2000) Tau protein isoforms, phosphorylation and role in neurodegenerative disorders. Brain Res. Rev. 33, 95–130 10.1016/S0165-0173(00)00019-9 10967355

[43] Binder L.I., Guillozet-Bongaarts A.L., Garcia-Sierra F. and Berry R.W. (2005) Tau, tangles, and Alzheimer’s disease. Biochim. Biophys. Acta Mol. Basis Dis. 1739, 216–223 10.1016/j.bbadis.2004.08.014 15615640

[44] Forman M.S., Zhukareva V., Bergeron C., Chin S.S.M., Grossman M., Clark C. et al. (2002) Signature tau neuropathology in gray and white matter of corticobasal degeneration. Am. J. Pathol. 160, 2045–2053 10.1016/S0002-9440(10)61154-6 12057909 PMC1850831

[45] Wegmann S., Eftekharzadeh B., Tepper K., Zoltowska K.M., Bennett R.E., Dujardin S. et al. (2018) Tau protein liquid-liquid phase separation can initiate tau aggregation. EMBO J. 37, e98049 10.15252/embj.201798049 29472250 PMC5881631

[46] Boyko S. and Surewicz W.K. (2022) Tau liquid-liquid phase separation in neurodegenerative diseases. Trends Cell Biol. 32, 611–623 10.1016/j.tcb.2022.01.011 35181198 PMC9189016

[47] Kanaan N.M., Hamel C., Grabinski T. and Combs B. (2020) Liquid-liquid phase separation induces pathogenic tau conformations *in vitro*. Nat. Commun. 11, 2809 10.1038/s41467-020-16580-3 32499559 PMC7272632

[48] Yang J., Shen N., Shen J., Yang Y. and Li H.-L. (2024) Complicated role of post-translational modification and protease-cleaved fragments of tau in Alzheimer’s disease and other tauopathies. Mol. Neurobiol. 61, 4712–4731 10.1007/s12035-023-03867-x 38114762 PMC11236937

[49] Kyalu Ngoie Zola N., Balty C., Pyr dit Ruys S., Vanparys A.A.T., Huyghe N.D.G., Herinckx G. et al. (2023) Specific post-translational modifications of soluble tau protein distinguishes Alzheimer’s disease and primary tauopathies. Nat. Commun. 14, 3706 10.1038/s41467-023-39328-1 37349319 PMC10287718

[50] Alquezar C., Arya S. and Kao A.W. (2021) Tau post-translational modifications: dynamic transformers of tau function, degradation, and aggregation. Front. Neurol. 11, 595532 10.3389/fneur.2020.595532 33488497 PMC7817643

[51] Martin L., Latypova X. and Terro F. (2011) Post-translational modifications of tau protein: implications for Alzheimer’s disease. Neurochem. Int. 58, 458–471 10.1016/j.neuint.2010.12.023 21215781

[52] Correa Marrero M., Mello V.H., Sartori P. and Beltrao P. (2025) Global comparative structural analysis of responses to protein phosphorylation. Nat. Commun. 16, 9407 10.1038/s41467-025-64116-4 41136353 PMC12552441

[53] Park E., Rawson S., Schmoker A., Kim B.W., Oh S., Song K. et al. (2023) Cryo-EM structure of a RAS/RAF recruitment complex. Nat. Commun. 14, 4580 10.1038/s41467-023-40299-6 37516774 PMC10387098

[54] Martinez Fiesco J.A., Beilina A., Alvarez de la Cruz A., Li N., Metcalfe R.D., Cookson M.R. et al. (2025) 14-3-3 binding maintains the Parkinson’s associated kinase LRRK2 in an inactive state. Nat. Commun. 16, 7226 10.1038/s41467-025-62337-1 40764514 PMC12325948

[55] Nadel C.M., Pokhrel S., Wucherer K., Oehler A., Thwin A.C., Basu K. et al. (2024) Phosphorylation of tau at a single residue inhibits binding to the E3 ubiquitin ligase, CHIP. Nat. Commun. 15, 7972 10.1038/s41467-024-52075-1 39266525 PMC11393453

[56] Rrustemi T., Meyer K., Roske Y., Uyar B., Akalin A., Imami K. et al. (2024) Pathogenic mutations of human phosphorylation sites affect protein-protein interactions. Nat. Commun. 15, 3146 10.1038/s41467-024-46794-8 38605029 PMC11009412

[57] Chai F., Xu W., Musoke T., Tarabelsi G., Assaad S., Freedman J. et al. (2019) Structure-function analysis of β-arrestin Kurtz reveals a critical role of receptor interactions in downregulation of GPCR signaling in vivo. Dev. Biol. 455, 409–419 10.1016/j.ydbio.2019.07.013 31325455 PMC6842422

[58] Eick D. and Geyer M. (2013) The RNA Polymerase II Carboxy-Terminal Domain (CTD) Code. Chem. Rev. 113, 8456–8490 10.1021/cr400071f 23952966

[59] Buratowski S. (2009) Progression through the RNA polymerase II CTD cycle. Mol. Cell 36, 541–546 10.1016/j.molcel.2009.10.019 19941815 PMC3232742

[60] Samarasimhareddy M., Mayer G., Hurevich M. and Friedler A. (2020) Multiphosphorylated peptides: importance, synthetic strategies, and applications for studying biological mechanisms. Organic & Biomolecular Chem. 18, 3405–3422 10.1039/D0OB00499E 32322853

[61] Kozelekova A., Naplavova A., Brom T., Gasparik N., Simek J., Houser J. et al. (2022) Phosphorylated and phosphomimicking variants may differ—a case study of 14-3-3 protein. Front. Chem. 10, 835733 10.3389/fchem.2022.835733 35321476 PMC8935074

[62] Thorsness P.E. and Koshland D.E. (1987) Inactivation of isocitrate dehydrogenase by phosphorylation is mediated by the negative charge of the phosphate. J. Biol. Chem. 262, 10422–10425 10.1016/S0021-9258(18)60975-5 3112144

[63] Mansour S.J., Matten W.T., Hermann A.S., Candia J.M., Rong S., Fukasawa K. et al. (1994) Transformation of mammalian cells by constitutively active MAP kinase kinase. Science 265, 966–970 10.1126/science.8052857 8052857

[64] Pearlman S.M., Serber Z. and Ferrell J.E. Jr (2011) A mechanism for the evolution of phosphorylation sites. Cell 147, 934–946 10.1016/j.cell.2011.08.052 22078888 PMC3220604

[65] Otto N.M., McDowell W.G., Dickey D.M. and Potter L.R. (2017) A glutamate-substituted mutant mimics the phosphorylated and active form of guanylyl cyclase-A. Mol. Pharmacol. 92, 67–74 10.1124/mol.116.107995 28416574 PMC5452060

[66] Paleologou K.E., Schmid A.W., Rospigliosi C.C., Kim H.-Y., Lamberto G.R., Fredenburg R.A. et al. (2008) Phosphorylation at Ser-129 but not the phosphomimics S129E/D inhibits the fibrillation of α-synuclein*. J. Biol. Chem. 283, 16895–16905 10.1074/jbc.M800747200 18343814 PMC2423264

[67] Macdonald A., Campbell D.G., Toth R., McLauchlan H., Hastie C.J. and Arthur J.S. (2006) Pim kinases phosphorylate multiple sites on Bad and promote 14-3-3 binding and dissociation from Bcl-XL. BMC Cell Biol. 7, 1 10.1186/1471-2121-7-1 16403219 PMC1368972

[68] Kaneko T., Joshi R., Feller S.M. and Li S.S. (2012) Phosphotyrosine recognition domains: the typical, the atypical and the versatile. Cell Commun. Signal. 10, 32 10.1186/1478-811X-10-32 23134684 PMC3507883

[69] Song L., Liu Z., Hu H.H., Yang Y., Li T.Y., Lin Z.Z. et al. (2020) Proto-oncogene Src links lipogenesis via lipin-1 to breast cancer malignancy. Nat. Commun. 11, 5842 10.1038/s41467-020-19694-w 33203880 PMC7672079

[70] Conti M.M., Li R., Narvaez Ramos M.A., Zhu L.J., Fazzio T.G. and Benanti J.A. (2023) Phosphosite scanning reveals a complex phosphorylation code underlying CDK-dependent activation of Hcm1. Nat. Commun. 14, 310 10.1038/s41467-023-36035-9 36658165 PMC9852432

[71] Martinez-Lopez N., Mattar P., Toledo M., Bains H., Kalyani M., Aoun M.L. et al. (2023) mTORC2–NDRG1–CDC42 axis couples fasting to mitochondrial fission. Nat. Cell Biol. 25, 989–1003 10.1038/s41556-023-01163-3 37386153 PMC10344787

[72] Panda S.P., Gao Y.T., Roman L.J., Martásek P., Salerno J.C. and Masters B.S.S. (2006) The role of a conserved serine residue within hydrogen bonding distance of FAD in redox properties and the modulation of catalysis by Ca^2+^/calmodulin of constitutive nitric-oxide synthases. J. Biol. Chem. 281, 34246–34257 10.1074/jbc.M601041200 16966328

[73] Wan W.-Y. and Milner-White E.J. (1999) A recurring two-hydrogen-bond motif incorporating a serine or threonine residue is found both at α-helical N termini and in other situations. J. Mol. Biol. 286, 1651–1662 10.1006/jmbi.1999.2551 10064721

[74] Chin J.W. (2017) Expanding and reprogramming the genetic code. Nature 550, 53–60 10.1038/nature24031 28980641

[75] Ding W., Yu W., Chen Y., Lao L., Fang Y., Fang C. et al. (2024) Rare codon recoding for efficient noncanonical amino acid incorporation in mammalian cells. Science 384, 1134–1142 10.1126/science.adm8143 38843324

[76] Costello A., Peterson A.A., Lanster D.L., Li Z., Carver G.D. and Badran A.H. (2025) Efficient genetic code expansion without host genome modifications. Nat. Biotechnol. 43, 1116–1127 10.1038/s41587-024-02385-y 39261591

[77] Huang Y., Zhang P., Wang H., Chen Y., Liu T. and Luo X. (2025) Genetic code expansion: recent developments and emerging applications. Chem. Rev. 125, 523–598 10.1021/acs.chemrev.4c00216 39737807 PMC11758808

[78] Allen M.C., Karplus P.A., Mehl R.A. and Cooley R.B. (2024) Genetic encoding of phosphorylated amino acids into proteins. Chem. Rev. 124, 6592–6642 10.1021/acs.chemrev.4c00110 38691379 PMC11658404

[79] Park H.-S., Hohn M.J., Umehara T., Guo L.-T., Osborne E.M., Benner J. et al. (2011) Expanding the genetic code of *Escherichia coli* with phosphoserine. Science 333, 1151–1154 10.1126/science.1207203 21868676 PMC5547737

[80] Zhang M.S., Brunner S.F., Huguenin-Dezot N., Liang A.D., Schmied W.H., Rogerson D.T. et al. (2017) Biosynthesis and genetic encoding of phosphothreonine through parallel selection and deep sequencing. Nat. Methods 14, 729–736 10.1038/nmeth.4302 28553966 PMC5493988

[81] Arslan T., Mamaev S.V., Mamaeva N.V. and Hecht S.M. (1997) Structurally modified firefly luciferase. Effects of amino acid substitution at position 286. J. Am. Chem. Soc. 119, 10877–10887 10.1021/ja971927a

[82] Tarrant M.K. and Cole P.A. (2009) The chemical biology of protein phosphorylation. Annu. Rev. Biochem. 78, 797–825 10.1146/annurev.biochem.78.070907.103047 19489734 PMC3074175

[83] Barber K.W., Muir P., Szeligowski R.V., Rogulina S., Gerstein M., Sampson J.R. et al. (2018) Encoding human serine phosphopeptides in bacteria for proteome-wide identification of phosphorylation-dependent interactions. Nat. Biotechnol. 36, 638–644 10.1038/nbt.4150 29889213 PMC6590076

[84] Johnson S.A. and Hunter T. (2005) Kinomics: methods for deciphering the kinome. Nat. Methods 2, 17–25 10.1038/nmeth731 15789031

[85] Meng J.X., Zhang Y., Saman D., Haider A.M., De S., Sang J.C. et al. (2022) Hyperphosphorylated tau self-assembles into amorphous aggregates eliciting TLR4-dependent responses. Nat. Commun. 13, 2692 10.1038/s41467-022-30461-x 35577786 PMC9110413

[86] Piemontese E., Herfort A., Perevedentseva Y., Möller H.M. and Seitz O. (2024) Multiphosphorylation-dependent recognition of Anti-pS2 antibodies against RNA polymerase II C-terminal domain revealed by chemical synthesis. J. Am. Chem. Soc. 146, 12074–12086 10.1021/jacs.4c01902 38639141 PMC11066871

[87] Huse M., Holford M.N., Kuriyan J. and Muir T.W. (2000) Semisynthesis of hyperphosphorylated type I TGFβ receptor: addressing the mechanism of kinase activation. J. Am. Chem. Soc. 122, 8337–8338 10.1021/ja001763p

[88] Chiang K.P., Jensen M.S., McGinty R.K. and Muir T.W. (2009) A semi-synthetic strategy to generate phosphorylated and acetylated histone H2B. ChemBioChem 10, 2182 10.1002/cbic.200900238 19623598 PMC3086596

[89] Haj-Yahya M. and Lashuel H.A. (2018) Protein semisynthesis provides access to tau disease-associated post-translational modifications (PTMs) and paves the way to deciphering the tau PTM code in health and diseased states. J. Am. Chem. Soc. 140, 6611–6621 10.1021/jacs.8b02668 29684271

[90] Ellmer D., Brehs M., Haj‐Yahya M., Lashuel H.A. and Becker C.F. (2019) Single posttranslational modifications in the central repeat domains of Tau4 impact its aggregation and tubulin binding. Angew. Chem. Int. Ed. Engl. 131, 1630–1634 10.1002/ange.201805238 PMC6391969 30549369

[91] Dawson P.E., Muir T.W., Clark-Lewis I. and Kent S.B.H. (1994) Synthesis of proteins by native chemical ligation. Science 266, 776–779 10.1126/science.7973629 7973629

[92] Sánchez-Campillo I. and Blanco-Canosa J.B. (2024) Kinetic and mechanistic studies of native chemical ligation with phenyl α-selenoester peptides. JACS Au 4, 4374–4382 10.1021/jacsau.4c00705 39610746 PMC11600164

[93] Canne L.E., Bark S.J. and Kent S.B.H. (1996) Extending the applicability of native chemical ligation. J. Am. Chem. Soc. 118, 5891–5896 10.1021/ja960398s

[94] Dawson P.E., Churchill M.J., Ghadiri M.R. and Kent S.B.H. (1997) Modulation of reactivity in native chemical ligation through the use of Thiol additives. J. Am. Chem. Soc. 119, 4325–4329 10.1021/ja962656r

[95] Li T., Liu H. and Li X. (2016) Chemical synthesis of HMGA1a proteins with post-translational modifications via Ser/Thr ligation. Org. Lett. 18, 5944–5947 10.1021/acs.orglett.6b03056 27934496

[96] Mende F. and Seitz O. (2011) 9‐Fluorenylmethoxycarbonyl‐based solid‐phase synthesis of peptide α‐thioesters. Angew. Chem. Int. Ed. Engl. 50, 1232–1240 10.1002/anie.201005180 21290490

[97] Kawakami T., Hasegawa K., Teruya K., Akaji K., Horiuchi M., Inagaki F. et al. (2001) Polypeptide synthesis using an expressed peptide as a building block for condensation with a peptide thioester: application to the synthesis of phosphorylated p21Max protein (1–101). J. Pept. Sci. 7, 474–487 10.1002/psc.341 11587186

[98] Schrems M., Kravchuk A.V., Niederacher G., Exler F., Bello C. and Becker C.F.W. (2023) Light-cleavable auxiliary for diselenide-selenoester ligations of peptides and proteins. Chemistry 29, e202301253 10.1002/chem.202301253 37265454 PMC10946927

[99] Trunschke S., Piemontese E., Fuchs O., Abboud S. and Seitz O. (2022) Enhancing auxiliary‐mediated native chemical ligation at challenging junctions with pyridine scaffolds. Chemistry 28, 10.1002/chem.202202065 PMC10091703 36097325

[100] Sun Z., Liu H. and Li X. (2024) Precision in protein chemical modification and total synthesis. CCS Chem. 10, 767–799 10.1016/j.chempr.2023.10.020

[101] Han J., Hirao K., Mikami T., Nötel N.Y., Seidl L.L. and Bode J.W. (2025) Cyclic hydroxylamines for native residue-forming peptide ligations: synthesis of ubiquitin and tirzepatide. J. Am. Chem. Soc. 147, 34238–34243 10.1021/jacs.5c11881 40934092 PMC12464992

[102] Zou Z., Ji Y. and Schwaneberg U. (2024) Empowering site-specific bioconjugations *in vitro* and *in vivo*: advances in sortase engineering and sortase-mediated ligation. Angew. Chem. Int. Ed. Engl. 63, e202310910 10.1002/anie.202310910 38081121

[103] Braga Emidio N. and Cheloha R.W. (2024) Sortase-mediated labeling: expanding frontiers in site-specific protein functionalization opens new research avenues. Curr. Opin. Chem. Biol. 80, 102443 10.1016/j.cbpa.2024.102443 38503199 PMC11164631

[104] Staus D.P., Wingler L.M., Choi M., Pani B., Manglik A., Kruse A.C. et al. (2018) Sortase ligation enables homogeneous GPCR phosphorylation to reveal diversity in β-arrestin coupling. Proc. Natl. Acad. Sci. U.S.A. 115, 3834–3839 10.1073/pnas.1722336115 29581292 PMC5899476

[105] Kochinyan S., Sun L., Ghosh I., Barshevsky T., Xu J. and Xu M.-Q. (2007) Use of intein-mediated phosphoprotein arrays to study substrate specificity of protein phosphatases. BioTechniques 42, 63–69 10.2144/000112311 17269486

[106] Xu L., Zhang Y., Li Y.-M. and Lu X.-F. (2020) Total chemical synthesis of the phosphorylated p62 UBA domain reveals that Ser 407 Pi but not Ser 403 Pi enhances ubiquitin binding. Org. Biomol. Chem. 18, 8709–8715 10.1039/D0OB01906B 33084718

[107] Pan M., Zheng Q., Gao S., Qu Q., Yu Y., Wu M. et al. (2019) Chemical synthesis of structurally defined phosphorylated ubiquitins suggests impaired Parkin activation by phosphorylated ubiquitins with a non-phosphorylated distal unit. CCS Chem. 1, 476–489 10.31635/ccschem.019.20190001

[108] Harmand T.J., Pattabiraman V.R. and Bode J.W. (2017) Chemical synthesis of the highly hydrophobic antiviral membrane‐associated protein IFITM3 and modified variants. Angew. Chem. Int. Ed. Engl. 56, 12639–12643 10.1002/anie.201707554 28834009 PMC5658968

[109] Pihl R., Zheng Q. and David Y. (2023) Nature-inspired protein ligation and its applications. Nat. Rev. Chem. 7, 234–255 10.1038/s41570-023-00468-z 37117416 PMC10659114

[110] Morgan H.E., Turnbull W.B. and Webb M.E. (2022) Challenges in the use of sortase and other peptide ligases for site-specific protein modification. Chem. Soc. Rev. 51, 4121–4145 10.1039/D0CS01148G 35510539 PMC9126251

[111] Freund C. and Schwarzer D. (2021) Engineered sortases in peptide and protein chemistry. ChemBioChem 22, 1347–1356 10.1002/cbic.202000745 33290621 PMC8248031

[112] Despres C., Byrne C., Qi H., Cantrelle F.X., Huvent I., Chambraud B. et al. (2017) Identification of the Tau phosphorylation pattern that drives its aggregation. Proc. Natl. Acad. Sci. U.S.A. 114, 9080–9085 10.1073/pnas.1708448114 28784767 PMC5576827

[113] Wu C., Ba Q., Lu D., Li W., Salovska B., Hou P. et al. (2021) Global and site-specific effect of phosphorylation on protein turnover. Dev. Cell 56, 111–124 10.1016/j.devcel.2020.10.025 33238149 PMC7855865

[114] Vanova V., Mitrevska K., Milosavljevic V., Hynek D., Richtera L. and Adam V. (2021) Peptide-based electrochemical biosensors utilized for protein detection. Biosens. Bioelectron. 180, 113087 10.1016/j.bios.2021.113087 33662844

[115] Marx H., Lemeer S., Schliep J.E., Matheron L., Mohammed S., Cox J. et al. (2013) A large synthetic peptide and phosphopeptide reference library for mass spectrometry-based proteomics. Nat. Biotechnol. 31, 557–564 10.1038/nbt.2585 23685481

[116] Hoermann B., Kokot T., Helm D., Heinzlmeir S., Chojnacki J.E., Schubert T. et al. (2020) Dissecting the sequence determinants for dephosphorylation by the catalytic subunits of phosphatases PP1 and PP2A. Nat. Commun. 11, 10.1038/s41467-020-17334-x 32681005 PMC7367873

[117] Zhang Y., Kim Y., Genoud N., Gao J., Kelly J.W., Pfaff S.L. et al. (2006) Determinants for dephosphorylation of the RNA polymerase II C-terminal domain by Scp1. Mol. Cell 24, 759–770 10.1016/j.molcel.2006.10.027 17157258 PMC2859291

[118] Mühlbacher W., Mayer A., Sun M., Remmert M., Cheung A.C., Niesser J. et al. (2015) Structure of Ctk3, a subunit of the RNA polymerase II CTD kinase complex, reveals a noncanonical CTD-interacting domain fold. Proteins 83, 1849–1858 10.1002/prot.24869 26219431

[119] Cardasis H.L., Sehnke P.C., Laughner B., Eyler J.R., Powell D.H. and Ferl R.J. (2007) FTICR-MS analysis of 14-3-3 isoform substrate selection. Biochim. Biophys. Acta 1774, 866–873 10.1016/j.bbapap.2007.05.004 17569603

[120] Eissler C.L., Bremmer S.C., Martinez J.S., Parker L.L., Charbonneau H. and Hall M.C. (2011) A general strategy for studying multisite protein phosphorylation using label-free selected reaction monitoring mass spectrometry. Anal. Biochem. 418, 267–275 10.1016/j.ab.2011.07.015 21810403 PMC3172394

[121] Ablorh N.A., Dong X., James Z.M., Xiong Q., Zhang J., Thomas D.D. et al. (2014) Synthetic phosphopeptides enable quantitation of the content and function of the four phosphorylation states of phospholamban in cardiac muscle. J. Biol. Chem. 289, 29397–29405 10.1074/jbc.M114.556621 25190804 PMC4200288

[122] McMurray J.S., Coleman D.R., Wang W. and Campbell M.L. (2001) The synthesis of phosphopeptides. Biopolymers 60, 3–31 10.1002/1097-0282(2001)60:1<3::AID-BIP1001>3.0.CO;2-L 11376430

[123] Attard T.J., O'Brien-Simpson N. and Reynolds E.C. (2007) Synthesis of phosphopeptides in the Fmoc mode. Int. J. Pept. Res. Ther. 13, 447–468 10.1007/s10989-007-9107-y

[124] Perich J.W. and Johns R. (1988) Di-t-butyl N,N-diethylphosphoramidite and dibenzyl N,N-diethylphosphoramidite. Highly reactive reagents for the ‘phosphite-triester’phosphorylation of serine-containing peptides. Tetrahedron Lett. 29, 2369–2372 10.1016/S0040-4039(00)86062-1

[125] Arendt A., Palczewski K., Moore W.T., Caprioli R.M., McDowell J.H. and Hargrave P.A. (1989) Synthesis of phosphopeptides containing O‐phosphoserine or O‐phosphothreonine. Int. J. Pept. Protein Res. 33, 468–476 10.1111/j.1399-3011.1989.tb00225.x 2506143

[126] Petrillo D.E., Mowrey D.R., Allwein S.P. and Bakale R.P. (2012) A general preparation of protected phosphoamino acids. Org. Lett. 14, 1206–1209 10.1021/ol203332j 22356680

[127] Mowrey D.R., Petrillo D.E., Allwein S.P., Graf Sp. and Bakale R.P. (2012) Demonstration of a scalable one-pot synthesis of Fmoc-O-Benzylphospho-l-serine. Org. Process Res. Dev. 16, 1861–1865 10.1021/op300233g

[128] Attard T.J., O'Brien-Simpson N.M. and Reynolds E.C. (2009) Identification and suppression of β-elimination byproducts arising from the use of Fmoc-Ser(PO3Bzl,H)-OH in peptide synthesis. Int. J. Pept. Res. Ther. 15, 69–79 10.1007/s10989-008-9165-9

[129] Harris P.W.R., Williams G.M., Shepherd P. and Brimble M.A. (2008) The synthesis of phosphopeptides using microwave-assisted solid phase peptide synthesis. Int. J. Pept. Res. Ther. 14, 387–392 10.1007/s10989-008-9149-9

[130] Williams E.T., Schiefelbein K., Schuster M., Ahmed I.M., De Vries M., Beveridge R. et al. (2024) Rapid flow-based synthesis of post-translationally modified peptides and proteins: a case study on MYC’s transactivation domain. Chem. Sci. 15, 8756–8765 10.1039/D4SC00481G 38873065 PMC11168107

[131] Samarasimhareddy M., Mayer D., Metanis N., Veprintsev D., Hurevich M. and Friedler A. (2019) A targeted approach for the synthesis of multi-phosphorylated peptides: a tool for studying the role of phosphorylation patterns in proteins. Org. Biomol. Chem. 17, 9284–9290 10.1039/C9OB01874C 31497840

[132] Grunhaus D., Friedler A. and Hurevich M. (2021) Automated synthesis of heavily phosphorylated peptides. Eur. J. Org. Chem. 2021, 3737–3742 10.1002/ejoc.202100691

[133] Grunhaus D., Molina E.R., Cohen R., Stein T., Friedler A. and Hurevich M. (2022) Accelerated multiphosphorylated peptide synthesis. Org. Process Res. Dev. 26, 2492–2497 10.1021/acs.oprd.2c00164 36032360 PMC9397535

[134] Bakhatan Y., Alshanski I., Chan C.K., Lo W.C., Lu P.W., Liao P.H. et al. (2023) Accelerated solid phase glycan synthesis: ASGS. Chemistry 29, e202300897 10.1002/chem.202300897 37035910

[135] Naoum J.N., Alshanski I., Mayer G., Strauss P. and Hurevich M. (2022) Stirring peptide synthesis to a new level of efficiency. Org. Process Res. Dev. 26, 129–136 10.1021/acs.oprd.1c00304

[136] Alshanski I., Bentolila M., Gitlin-Domagalska A., Zamir D., Zorsky S., Joubran S. et al. (2018) Enhancing the efficiency of the solid phase peptide synthesis (SPPS) process by high shear mixing. Org. Process Res. Dev. 22, 1318–1322 10.1021/acs.oprd.8b00225

[137] Kliche J., Garvanska D.H., Simonetti L., Badgujar D., Dobritzsch D., Nilsson J. et al. (2023) Large-scale phosphomimetic screening identifies phospho-modulated motif-based protein interactions. Mol. Syst. Biol. 19, e11164 10.15252/msb.202211164 37219487 PMC10333884

[138] Zhu P., Mehl R.A. and Cooley R.B. (2022) Site-specific incorporation of phosphoserine into recombinant proteins in *Escherichia coli*. Bio. Protoc. 12, 10.21769/BioProtoc.4541 PMC9711932 36505030

[139] von Bergen M., Friedhoff P., Biernat J., Heberle J., Mandelkow E.M. and Mandelkow E. (2000) Assembly of tau protein into Alzheimer paired helical filaments depends on a local sequence motif ((306)VQIVYK(311)) forming beta structure. Proc. Natl. Acad. Sci. U.S.A. 97, 5129 10.1073/pnas.97.10.5129 10805776 PMC25793

[140] Li W. and Lee V.M. (2006) Characterization of two VQIXXK motifs for tau fibrillization *in vitro*. Biochemistry 45, 15692 10.1021/bi061422 17176091

[141] Bressler S.G., Grunhaus D., Aviram A., Rudiger S.G.D., Hurevich M. and Friedler A. (2025) Specific phosphorylation patterns control the interplay between aggregation and condensation of Tau-R4 peptides. Org. Biomol. Chem. 10.1039/D5OB00885A 40567043

